# Incidence and severity of SARS-CoV-2 infection in children and young people with HIV in Europe

**DOI:** 10.1097/QAD.0000000000003610

**Published:** 2023-05-29

## Abstract

We assessed incidence of SARS-CoV-2 infection and disease severity among children and young people with HIV from cohorts across nine European countries. Of 1717 included, with median duration of follow-up 20.1 months, 134 (8%) had documented SARS-CoV-2 infection, a rate of 49 [95% confidence interval (CI) 42–58] per 1000 person-years. All symptomatic cases had mild coronavirus disease 2019 (COVID-19), three were hospitalized, and no deaths were reported, which may be reassuring for clinicians and families.

In adult studies HIV infection has been independently associated with poorer outcomes of coronavirus disease 2019 (COVID-19) [1–3]; however, there remains limited data in children and young people with HIV (CYPHIV).

We estimate the incidence of SARS-CoV-2 infection in CYPHIV in the European Pregnancy and Paediatric Infections Cohort Collaboration (EPPICC) and describe the clinical characteristics of those with COVID-19.

EPPICC collects data on CYPHIV in routine care [4]. CYPHIV are followed from entry to HIV care to last visit in paediatric care, or in some cohorts through adult care. CYPHIV from 11 cohorts in 9 European countries who were diagnosed with HIV aged <18 years and in follow-up aged<25 years on/after 1 January 2020 (proxy for start of pandemic in Europe) were included.

SARS-CoV-2 infection was defined as documented positive PCR test based on clinical notes or linkage with national/regional SARS-CoV-2 databases. Multisystem Inflammatory Syndrome in Children (MIS-C) cases (WHO/ISARIC case definition [5]) were requested, but none identified.

Pseudonymized individual-level data were pooled using a modified HIV Cohorts Data Exchange Protocol (www.hicdep.org) and a standardized case report form based on WHO/ISARIC forms [5] to collect additional data on those with SARS-CoV-2 infection. All cohorts received local ethics approval where required.

Individuals were considered at risk from latest of 1 January 2020, and date of birth for those with vertically-acquired HIV or date first seen in HIV care for those with other modes of acquisition. Follow-up was censored at SARS-CoV-2 diagnosis, death, age 25 years or last visit before data cut-off.

Among those in HIV care at start of 2020, characteristics on 1 January 2020 were compared between those with and without SARS-CoV-2 infection, using chi-squared tests for categorical and Wilcoxon's rank-sum tests for continuous variables. COVID-19 severity was classified using WHO criteria [6].

Among 1717 CYPHIV included, the median [interquartile range (IQR)] duration of follow-up was 20.1 months (14.1–24.6), and median age on 1 January 2020 was 13.9 (9.1–17.8) years. One hundred and thirty-four (8%) were diagnosed with SARS-CoV-2 infection, an incidence rate of 49 (95% confidence interval 42–58) per 1000 person-years.

Among the 1688 CYPHIV in follow-up on 1 January 2020, those with SARS-CoV-2 infection were older than those without, had been diagnosed with HIV for a longer time, and were less likely to have an HIV RNA viral load<50 copies/ml (all *P* < 0.06), with no difference in CD4 count, antiretroviral treatment (ART) status or obesity (all *P* > 0.35). Of the 134 with SARS-CoV-2, 63 (47%) had known date of diagnosis: median age at diagnosis was 17.0 years, at which point 57/63 (90%) were on ART (Table 1).

**Table 1:** Demographic and HIV-related clinical characteristics of participants by SARS-CoV-2 infection status.

	SARS-CoV-2 infection (*N* = 134)	No SARS-CoV-2 infection (*N* = 1583)	*P*
	*n* (%) or median (IQR)		
**Demographics**			
Female sex	79 (59%)	871 (55%)	0.379
Ethnicity (*n* = 133; 1570)			0.439
White	97 (73%)	1063 (68%)	
Black	29 (22%)	420 (27%)	
Other	7 (5%)	87 (6%)	
Vertically acquired HIV (*n* = 113; 1367)	101 (89%)	1272 (93%)	0.148
Country			<0.001
Belgium	1 (1%)	58 (4%)	
Greece	0	21 (1%)	
Poland	1 (1%)	44 (3%)	
Portugal	1 (1%)	23 (1%)	
Romania	0	54 (3%)	
Russia	84 (65%)	841 (53%)	
Spain	23 (17%)	159 (10%)	
Sweden	0	113 (7%)	
UK	24 (19%)	270 (17%)	
**Characteristics on 1 January 2020 (*n* = 130 with SARS-CoV-2 infection and *n* = 1558 without)** ^a^			
Age, years	15.8 (11.7, 18.8)	13.7 (8.9, 17.7)	0.002
Years since HIV diagnosis (*n* = 126; 1483)	10.9 (4.8, 15.1)	9.1 (4.6, 13.2)	0.050
CD4^+^ cell count in those ≥5 years (*n* = 109; 1276)	713 (537, 916)	730 (531, 980)	0.456
CD4^+^% in those <5 years (*n* = 7; 144)	39 (30, 44)	37 (32, 44)	0.953
WHO advanced/severe immunosuppression for age^b^ (*n* = 116; 1420)	9 (8%)	119 (8%)	0.816
HIV viral load <50 copies/ml (*n* = 93; 1240)	45 (48%)	726 (59%)	0.056
ART status			0.835
ART naïve	6 (5%)	79 (5%)	
Off ART (>7 days)	7 (5%)	102 (7%)	
On ART	116 (89%)	1353 (87%)	
Unknown	1 (1%)	24 (2%)	
Obesity^c^ in those ≥2 years (*n* = 77; 1035)			0.350
Underweight/normal weight	59 (78%)	856 (83%)	
Overweight	14 (18%)	131 (13%)	
Obese	3 (4%)	48 (5%)	
**Characteristics at SARS-CoV-2 infection (*n* = 63 with date of infection known)**			
Age, years	17.0 (14.1, 19.5)	–	–
ART status		–	–
ART naïve	1 (2%)	–	–
Off ART (>7 days)	4 (6%)	–	–
On ART	57 (90%)	–	–
Unknown	1 (2%)	–	–
CD4^+^ count in those ≥5 years (*n* = 48)	721 (585, 973)	–	–
CD4^+^% in those <5 years (*n* = 3)	34 (33, 54)	–	–
HIV viral load <50 copies/ml (*n* = 50)	39 (78%)	–	–
Reason for PCR test		–	–
Contact tracing	16 (25%)	–	–
Presented symptomatically	11 (17%)	–	–
Routine screening	2 (3%)	–	–
Unknown	34 (54%)	–	–
Any symptoms experienced (among those with clinical data (*n* = 42)	25 (60%)	–	–
Cough	13 (31%)	–	–
Anosmia and/or aguesia	11 (26%)	–	–
Fever	11 (26%)	–	–
Headache	8 (19%)	–	–
Fatigue	7 (17%)	–	–
Runny nose	7 (17%)	–	–
Sore throat	7 (17%)	–	–

Only symptoms experienced by ≥10% of patients are listed in the table. Symptoms experienced by <10% of patients were: myalgia, vomiting, abdominal pain, diarrhoea, increased respiratory rate, joint pain, rash and wheezing. The ‘*n*’ given refers to the number with nonmissing data, with data being complete if not specified.

a Excludes 4/134 diagnosed with COVID and 25/1583 not diagnosed with SARS-CoV-2 infection who were born or entered care >1 January 2020.

b WHO advanced/severe immunosuppression for age defined as CD4^+^ lymphocyte percentage (CD4^+^%) <30% for children <1 year of age, <25% for children aged 1–3 years, <20% for children aged 3–5 years, and <650 cells/mm^3^ or <15% for children aged ≥5 years.

c Based on WHO BMI-for-age *z*-score: overweight corresponds to *z*-score ≥2, obese corresponds to *z*-score ≥3 [11,12].

Among 44/134 (33%) SARS-CoV-2 cases with clinical data available, 42 had symptom information, of whom 25 (60%) experienced ≥1 symptom of COVID-19. The most common symptoms were cough (13/42, 31%), fever (11/42, 26%), and anosmia and/or ageusia (11/42, 26%). COVID-19 was asymptomatic for 17 (39%), mild for 22 (50%) and unknown for 5 (11%); none had moderate, severe or critical disease.

Three individuals were hospitalized following SARS-CoV-2 diagnosis. The first was aged <10 years, recently diagnosed with HIV/AIDS, severely immunocompromised (CD4^+^ 6 cells/mm^3^) and had been on ART for <1 month. They were admitted for 23 days. Their symptoms included increased respiratory rate and wheezing. Chest X-ray was normal. They received oral fluids, interferon-alpha, ceftriaxone and ibuprofen; however, the primary reason for admission was not COVID-19. The second and third patients were older adolescents hospitalized for 1 and 3 days, respectively. Both had an HIV RNA viral load<50 copies/ml, were not significantly immunocompromised (CD4^+^ >350 cells/mm^3^), and had mild COVID-19.

Among the 44 patients with clinical data, 1 (3%) had ongoing symptoms at 6 months; they were not hospitalized, and their main ongoing symptom was fatigue. No deaths were reported.

This study represents the first multicountry description of SARS-CoV-2 infection and COVID-19 specifically among CYPHIV.

The incidence of diagnosed SARS-CoV-2 infection was relatively low, although the timeframe over which data were available varied by country, and in some cohorts only captured the early part of the pandemic. In the general population incidence of symptomatic COVID-19 among those <18 years across 10 European countries between August 2020 and September 2021 was estimated at 27 per 1000 population, lower than reported here [7]; however, comparison is difficult, given differences in setting, time period and case definition. Infection was more common among those who were older and those not virally-suppressed. An increased risk of infection with age is consistent with data from the general population [8].

Although this study includes relatively small numbers with SARS-CoV-2, there was no suggestion of increased risk of severe outcomes as observed in adult HIV populations, with none having WHO moderate/severe disease, few hospitalized and no deaths. A study from South Africa reported a two-fold increase in COVID-19 death among people aged ≥20 years with HIV compared to those without [1], although they were unable to adjust for key confounders such as comorbidities. The largest published study on people with HIV with COVID-19 is from the WHO Global COVID-19 clinical platform [2], which observed 15% higher odds of severe/critical presentation and 38% increased odds of death among patients with HIV compared to those without HIV, after adjusting for multiple confounders including comorbidities. However, the study was restricted to those hospitalized with COVID-19. The largest study specific to CYPHIV to date was of 60 individuals in a Spanish paediatric HIV cohort (included here), which reported 8 (13%) cases diagnosed with SARS-CoV-2 [9].

There are important study limitations. Firstly, availability of SARS-CoV-2 PCR testing varied across countries over time and asymptomatic infections were likely missed, leading to an underestimation of incidence. In comparison, a serology study of ∼500 participants from the EPPICC cohort reported 55% were seropositive by mid-2022 [10]. Further, no data on potentially ‘silent’ symptoms of MIS-C, such as myocarditis, were available. However, we are confident that severe cases would have been reported to HIV clinics and captured here, so our conclusion of SARS-CoV-2 infection being overwhelmingly mild in this cohort is likely correct. Secondly, dates of SARS-CoV-2 infection and clinical details were not available for all cases. Thirdly, small numbers of cases meant estimating rates of infection over time was not possible. Finally, data on SARS-CoV-2 vaccination were not collected, as data collection began before rollout.

## Acknowledgements

We thank all the patients for their participation in these cohorts, and the staff members who cared for them.

**Author contributors:** Writing Group [consisting of Project Team first (ordered by contribution), followed by other Writing Group members (ordered alphabetically by country)]:

Project Team: Elizabeth Chappell (EPPICC statistician, MRC Clinical Trials Unit at UCL, University College London, London, UK), Alasdair Bamford (Collaborative HIV Paediatric Study (CHIPS), UK; MRC Clinical Trials Unit at UCL, University College, London, London, UK; Great Ormond Street Hospital for Children NHS Trust, London, UK; University College London Great Ormond Street; Institute of Child Health, London, UK), Arantxa Berzosa Sanchez (CoRISPE-S, rest of Spain cohort, Spain; Pediatric Infectious Diseases Unit, Department of Pediatrics, Hospital Clínico San Carlos, Madrid, Spain; Health Research Institute of the Hospital Clínico San Carlos (IdISSC), Pediatric National AIDS Research, Network of Spain (CoRISpe), Madrid, Spain; Centro de Investigacion Biomedica en Red en Enfermedades Infecciosas, (CIBERINFEC), ISCIII, Madrid, Spain), Tessa Goetghebuer (Hospital St Pierre Cohort, Brussels, Belgium), Laura Marques (Centro Hospitalar do Porto, Porto, Portugal), Lars Navér (Karolinska University Hospital, Stockholm, Sweden), Antoni Noguera-Julian (CoRISPE-CAT, Catalonia cohort, Spain; Unitat d’Infectologia, Servei de Pediatria, Hospital Sant Joan de Deu, Universitat de Barcelona, Barcelona, Spain), Ewa Talarek (Polish paediatric cohort, Poland; Department of Children's Infectious Diseases, Medical University of Warsaw; Regional Hospital of Infectious Diseases in Warsaw, Poland), Yulia Plotnikova (Irkutsk AIDS Centre, Irkutsk, Russia), Anna Samarina (The City HIV Centre, St Petersburg, Russia), Luminita Ene (Victor Babes’ Hospital Cohort, Romania; Clinical Department of Infectious Diseases (HIV Department), Dr. Victor Babes¸ Hospital for Infectious and Tropical Diseases, Bucharest, Romania), Vana Spoulou (Greek cohort, Greece; “Agia Sophia” Children's Hospital, Paediatric HIV Unit, National and Kapodistrian University of Athens, Greece), Intira Jeannie Collins (co-lead of EPPICC; MRC Clinical Trials Unit at UCL, University College London, London, UK)

Other Writing Group members: Marisa Luisa Navarro (Red de Investigacíon Translacional en Infectología Pedíatrica, RITIP, Madrid, Spain; Hospital General Universitario “Gregorio Marañón”, Madrid, Spain; Universidad Complutense, Madrid, Spain; and Instituto de Investigaci_on Sanitaria Gregorio Marañón (IISGM), Spain), Aleksey A. Plynsky (Irkutsk AIDS Center, Irkutsk, Russia), Yulia Chapygina (Irkutsk AIDS Center, Irkutsk, Russia), Elena Fertikh (Saint Petersburg City HIV Centre, St. Petersburg, Russia), Sandra Soeria-Atmadja (Department of Paediatrics, Karolinska University Hospital, Stockholm, Sweden), Katja Doerholt (St George's University Hospital NHS Foundation Trust, London, UK), Steve Welch (Department of Paediatrics, Heartlands Hospital, Birmingham, UK), Siobhan Crichton (MRC Clinical Trials Unit at UCL, University College London, London, UK), Charlotte Jackson (MRC Clinical Trials Unit at UCL, University College London, London, UK), Ali Judd (MRC Clinical Trials Unit at UCL, University College London, London, UK), Karen Scott (MRC Clinical Trials Unit at UCL, University College London, London, UK)

**EPPICC/Penta Co-ordinating Team**: Elizabeth Chappell, Siobhan Crichton, Intira Jeannie Collins, Giorgia Dalla Valle, Charlotte Duff, Carlo Giaquinto, Charlotte Jackson, Ali Judd, Laura Mangiarini, Edith Milanzi, Karen Scott, Claire Thorne

**Collaborating cohorts:**

Belgium: Hopital St Pierre Cohort, Brussels: Tessa Goetghebuer, MD, PhD; Marc Hainaut, MD PhD; Wivine Tremerie, Research nurse; Marc Delforge, data manager.

Greece: Greek cohort: Vana Spoulou.

Poland: Polish paediatric cohort: Head of the team: Prof Magdalena Marczyńska, MD, PhD Members of the team: Jolanta Popielska, MD, PhD; Maria Pokorska-Śpiewak, MD, PhD; Agnieszka Ołdakowska, MD, PhD; Konrad Zawadka, MD, PhD; Magdalena Pluta MD, PhD Administration assistant: Małgorzata Doroba. Affiliation: Medical University of Warsaw, Poland, Department of Children's Infectious Diseases; Hospital of Infectious Diseases in Warsaw, Poland.

Portugal: Centro Hospitalar do Porto:Laura Marques, Carla Teixeira, Alexandre Fernandes.

Romania: ‘Victor Babes’ Hospital Cohort, Bucharest: Dr Luminita Ene.

Russia: The City HIV Centre, St Petersburg: Anna Samarina

Russia: Irkutsk AIDS Centre: Anna Turkova, Yulia Plotnikova

Spain: CoRISPE-cat, Catalonia: CoRISPE-cat receives financial support from the Instituto de Salud Carlos III through the Red Temática de Investigación Cooperativa en Sida (grant numbers RED RIS RD06/0006/0035 yRD06/0006/0021). Members: Hospital Universitari Vall d’Hebron, Barcelona (Pere Soler-Palacín, Maria Antoinette Frick and Santiago Pérez-Hoyos (statistician)), Hospital Universitari del Mar, Barcelona (Núria López), Hospital Universitari Germans Trias i Pujol, Badalona (María Méndez, Clara Carreras), Hospital Universitari JosepTrueta, Girona (Borja Guarch), Hospital Universitari Arnau de Vilanova, Lleida (Teresa Vallmanya, Laura Minguell-Domingo), Hospital Universitari Joan XXIII, Tarragona (Olga Calavia), Consorci Sanitari del Maresme, Mataró (Lourdes García), Hospital General de Granollers (Maite Coll), Corporació Sanitària Parc Taulí, Sabadell (Valentí Pineda), Hospital Universitari Sant Joan, Reus (Neus Rius), Fundació Althaia, Manresa (Núria Rovira), Hospital Son Espases, Mallorca (Joaquín Dueñas) and Hospital Sant Joan de Déu, Esplugues (Clàudia Fortuny, Anna Gamell, Antoni Noguera-Julian).

Spain: CoRISPE-S and Madrid cohort: *Receive funding from:* Estudio del análisis clínico-epidemiológico de la infección por el vih en niños y adolescentes, mujeres embarazadas y sus hijos a nivel nacional. Ministerio Sanidad. Proyect 202007PN0002.

*Paediatrics Units:* María José Mellado, Luis Escosa, Milagros García Hortelano, Talía Sainz, Carlos Grasa, Paula Rodríguez (Hospital Universitario La Paz, Madrid); Pablo Rojo, Luis Prieto-Tato, Cristina Epalza, Alfredo Tagarro, Sara Domínguez, Álvaro Ballesteros (Hospital Universitario Doce de Octubre, Madrid); José Tomás Ramos, Marta Illán, Arantxa Berzosa, (Hospital Clínico San Carlos, Madrid); Sara Guillén, Beatriz Soto (Hospital Universitario de Getafe, Madrid); María Luisa Navarro, Jesús Saavedra, Mar Santos, David Aguilera, Begoña Santiago, Santiago Jimenez de Ory (Hospital Universitario Gregorio Marañón, Madrid); Amanda Bermejo (Hospital Universitario de Móstoles, Madrid); María Penín (Hospital Universitario Príncipe de Asturias de Alcalá de Henares, Madrid); Jorge Martínez (Hospital Infantil Universitario Niño Jesús, Madrid); Katie Badillo (Hospital Universitario de Torrejón, Madrid); Ana Belén Jiménez (Hospital Fundación Jiménez Díaz, Madrid); Adriana Navas (Hospital Universitario Infanta Leonor, Madrid); Eider Oñate (Hospital Universitario Donostia, Guipúzcoa); Itziar Pocheville (Hospital Universitario Cruces, Vizcaya); Elisa Garrote (Hospital Universitario Basurto, Vizcaya); Elena Colino, Olga Afonso (Hospital Insular Materno Infantil, Gran Canaria); Jorge Gómez Sirvent (Hospital Universitario Virgen de la Candelaria, Tenerife); Mónica Garzón, Vicente Román (Hospital General, Lanzarote); Raquel Angulo (Hospital de Poniente de El Ejido, Almería); Olaf Neth, Lola Falcón (Hospital Universitario Virgen del Rocío, Sevilla); Pedro Terol (Hospital Universitario Virgen de la Macarena, Sevilla); Juan Luis Santos, Álvaro Vázquez (Hospital Universitario Virgen de las Nieves, Granada); Begoña Carazo, Antonio Medina (Hospital Regional Universitario, Málaga); Francisco Lendínez, Mercedes Ibáñez (Complejo Hospitalario Torrecárdenas, Almería); Estrella Peromingo, María Isabel Sánchez (Hospital Universitario Puerta del Mar, Cádiz); Beatriz Ruiz (Hospital Universitario Reina Sofía de Córdoba); Ana Grande (Complejo Hospitalario Universitario Infanta Cristina, Badajoz); Francisco José Romero (Complejo Hospitalario, Cáceres); Carlos Pérez, Alejandra Méndez (Hospital de Cabueñes, Asturias); Laura Calle (Hospital Universitario Central de Asturias); Marta Pareja (Complejo Hospitalario Universitario, Albacete); Begoña Losada (Hospital Virgen de la Salud, Toledo); Mercedes Herranz,(Hospital Virgen del Camino, Navarra); Matilde Bustillo (Hospital Universitario Miguel Servet, Zaragoza); Pilar Collado (Hospital Clínico Universitario Lozano Blesa, Zaragoza); José Antonio Couceiro (Complejo Hospitalario Universitario, Pontevedra); Leticia Vila (Complejo Hospitalario Universitario, La Coruña); Consuelo Calviño (Hospital Universitario Lucus Augusti, Lugo); Ana Isabel Piqueras, Manuel Oltra (Hospital Universitario La Fe, Valencia); César Gavilán (Hospital Universitario de San Juan de Alicante, Alicante); Elena Montesinos (Hospital General Universitario, Valencia); Marta Dapena (Hospital General, Castellón); Beatriz Jiménez (Hospital Universitario Marqués de Valdecilla, Cantabria); Ana Gloria Andrés (Complejo Hospitalario, León); Víctor Marugán, Carlos Ochoa (Complejo Hospitalario, Zamora); Ana Isabel Menasalvas, Eloísa Cervantes, Beatriz Álvarez (Hospital Universitario Virgen de la Arrixaca, Murcia) and Paediatric HIV-BioBank integrated in the Spanish AIDS Research Network and collaborating Centers.

*Adults Units:* Cristina Díez, (Hospital Universitario Gregorio Marañón, Madrid). Ignacio Bernardino, María Luisa Montes, Eulalia Valencia, Ana Delgado (Hospital Universitario La Paz, Madrid); Rafael Rubio, Federico Pulido, Otilia Bisbal (Hospital Universitario Doce de Octubre, Madrid); Alfonso Monereo Alonso (Hospital Universitario de Getafe, Madrid); Juan Berenguer, Cristina Díez, Teresa Aldamiz, Francisco Tejerina, Juan Carlos Bernaldo de Quirós, Belén Padilla, Raquel Carrillo, Pedro Montilla, Elena Bermúdez, Maricela Valerio (Hospital Universitario Gregorio Marañón, Madrid); Jose Sanz (Hospital Universitario Príncipe de Asturias de Alcalá de Henares, Madrid); Alejandra Gimeno (Hospital Universitario de Torrejon, Madrid); Miguel Cervero, Rafael Torres (Hospital Universitario Severo Ochoa de Leganés, Madrid); Santiago Moreno, María Jesús Perez, Santos del Campo (Hospital Universitario Ramon y Cajal, Madrid); Pablo Ryan, Jesus Troya (Hospital Universitario Infanta Leonor, Madrid); Jesus Sanz (Hospital Universitario La Princesa, Madrid); Juan Losa, Rafael Gomez (Hospital Universitario Fundacion Alcorcon, Madrid); Miguel Górgolas (Hospital Fundacion Jimenez Diaz, Madrid); Alberto Díaz, Sara de la Fuente (Hospital Universitario Puerta de Hierro de Majadahonda, Madrid); Jose Antonio Iribarren, Maria Jose Aramburu, Lourdes Martinez (Hospital Universitario Donostia, Guipuzcoa); Ane Josune Goikoetxea (Hospital Universitario Cruces, Vizcaya); Sofia Ibarra, Mireia de la Peña (Hospital Universitario Basurto, Vizcaya); Víctor Asensi (Hospital Universitario Central de Asturias); Michele Hernandez (Hospital Universitario Insular, Gran Canaria); María Remedios Alemán, Ricardo Pelazas, María del Mar Alonso, Ana María López, Dácil García, Jehovana Rodriguez (Hospital Universitario de Canarias, Tenerife); Miguel Angel Cardenes (Hospital Universitario Doctor Negrin, Gran Canaria); Manuel A. Castaño, Francisco Orihuela, Inés Pérez, Mª Isabel Mayorga (Hospital Regional Universitario, Málaga); Luis Fernando Lopez-Cortes, Cristina Roca, Silvia Llaves (Hospital Universitario Virgen del Rocio, Sevilla); Maria Jose Rios, Jesus Rodriguez, Virginia Palomo (Hospital Universitario Virgen de la Macarena, Sevilla); Juan Pasquau, Coral Garcia (Hospital Universitario Virgen de las Nieves, Granada); Jose Hernandez, Clara Martinez (Hospital Universitario Clinico San Cecilio, Granada); Antonio Rivero, Angela Camacho (Hospital Universitario Reina Sofia, Cordoba); Dolores Merino, Miguel Raffo, Laura Corpa (Hospital Universitario Juan Ramon Jimenez, Huelva); Elisa Martinez, Fernando Mateos, Jose Javier Blanch (Complejo Hospitalario Universitario, Albacete); Miguel Torralba (Hospital Universitario, Guadalajara); Piedad Arazo, Gloria Samperiz (Hospital Universitario Miguel Servet, Zaragoza); Celia Miralles, Antonio Ocampo, Guille Pousada (Hospital Alvaro Cunqueiro, Pontevedra); Alvaro Mena (Complejo Hospitalario Universitario, La Coruna); Marta Montero, Miguel Salavert, (Hospital Universitario La Fe, Valencia); Maria Jose Galindo, Natalia Pretel (Hospital Clinico Universitario, Valencia); Joaquín Portilla, Irene Portilla (Hospital General Universitario, Alicante); Felix Gutierrez, Mar Masia, Cati Robledano, Araceli Adsuar (Hospital General Universitario de Elche, Alicante); Carmen Hinojosa, Begoña Monteagudo (Hospital Clinico, Valladolid); Pablo Bachiller (Hospital General, Segovia); Jesica Abadía (Hospital Universitario Rio Hortega, Valladolid); Carlos Galera, Helena Albendin, Marian Fernandez (Hospital Universitario Virgen de la Arrixaca, Murcia); Jose Ramon Blanco (Complejo Hospitalario San Millan-San Pedro, la Rioja).

Sweden: Karolinska University Hospital, Stockholm, The Swedish InfCareHIV cohort (Lars Navér, Sandra Soeria-Atmadja, Erik Belfrage, Vendela Hagås).

UK: Collaborative HIV Paediatric Study (CHIPS): CHIPS is funded by the NHS (London Specialised Commissioning Group) and has received additional support from Abbott, Boehringer Ingelheim, Bristol-Myers Squibb, GlaxoSmithKline, Gilead Sciences, Janssen and Roche. The MRC Clinical Trials Unit at UCL is supported by the Medical Research Council (https://www.mrc.ac.uk) programme number MC_UU_00004/03.

*CHIPS Steering Committee:* Hermione Lyall (chair), Alasdair Bamford, Karina Butler, Katja Doerholt, Conor Doherty, Caroline Foster, Ian Harrison, Julia Kenny, Nigel Klein, Gillian Letting, Paddy McMaster, Fungai Murau, Edith Nsangi, Katia Prime, Andrew Riordan, Fiona Shackley, Delane Shingadia, Sharon Storey, Gareth Tudor-Williams, Anna Turkova, Steve Welch. MRC Clinical Trials Unit: Intira Jeannie Collins, Claire Cook, Siobhan Crichton, Donna Dobson, Keith Fairbrother, Diana M. Gibb, Ali Judd, Marthe Le Prevost, Nadine Van Looy. Integrated Screening Outcome Surveillance Service (ISOSS), UCL: Helen Peters, Kate Francis, Claire Thorne.

Hospitals participating in CHIPS in 2019/20: University Hospitals Birmingham NHS Foundation Trust, Birmingham: L. Thrasyvoulou, S. Welch; Brighton and Sussex University Hospitals NHS Trust: K. Fidler; University Hospitals Bristol NHS Foundation Trust, Bristol: J. Bernatoniene, F. Manyika; Calderdale and Huddersfield NHS Foundation Trust, Halifax: G. Sharpe; Derby Teaching Hospitals NHS Foundation Trust: B Subramaniam; Glasgow Royal Hospital for Children, Glasgow: R. Hague, V. Price; Great Ormond Street Hospital for Children NHS Foundation Trust, London: J Flynn, N. Klein, A Bamford, D. Shingadia, K Grant; Oxford University Hospitals NHS Foundation Trust, Oxford: S. Yeadon, S. Segal; King's College Hospital NHS Foundation Trust, London: S. Hawkins; Leeds Teaching Hospitals NHS Trust, Leeds: M. Dowie; University Hospitals of Leicester NHS Trust, Leicester: S. Bandi, E. Percival; Luton and Dunstable Hospital NHS Foundation Trust, Luton: M. Eisenhut; K. Duncan; Milton Keynes General University Hospital NHS Foundation Trust, Milton Keynes: L Anguvaa, L. Wren, Newcastle upon Tyne Hospitals NHS Foundation Trust, Newcastle: T. Flood, A. Pickering; The Pennine Acute Hospitals NHS Trust, Manchester: P. McMaster C Murphy; North Middlesex University Hospital NHS Trust, London: J. Daniels, Y Lees; Northampton General Hospital NHS Trust, Northampton: F Thompson; London North West Healthcare NHS Trust, Middlesex; A. Williams, B. Williams, S. Pope; Barts Health NHS trust, London Dr S. Libeschutz; Nottingham University Hospitals NHS Trust, Nottingham: L Cliffe, S. Southall; Portsmouth Hospitals NHS Trust, Portsmouth: A. Freeman; Raigmore Hospital, Inverness: H. Freeman; Royal Belfast Hospital for Sick Children, Belfast: S. Christie; Royal Berkshire NHS Foundation Trust, Reading: A. Gordon; Royal Children's Hospital, Aberdeen: D. Rosie Hague, L. Clarke; Royal Edinburgh Hospital for Sick Children, Edinburgh: L Jones, L Brown; Royal Free NHS Foundation Trust, London: M Greenberg; Alder Hey Children's NHS Foundation Trust, Liverpool: C. Benson, A. Riordan; Sheffield Children's NHS Foundation Trust, Sheffield: L. Ibberson, F Shackley; University Hospital Southampton NHS Foundation Trust, Southampton: S. Patel, J. Hancock; St George's University Hospitals NHS Foundation Trust, London: K. Doerholt, K Prime, M Sharland, S. Storey; Imperial College Healthcare NHS Trust, London: EGH Lyall, C. Foster, P. Seery, G. Tudor-Williams, N Kirkhope, S Raghunanan; Guy's and St Thomas’ NHS Foundation Trust, London: Dr Julia Kenny, A Callaghan; University Hospitals of North Midlands NHS Trust, Stoke On Trent: A Bridgwood, P McMaster; University Hospital of Wales, Cardiff: J. Evans, E. Blake; NHS Frimley Health Foundation Trust, Slough: A. Yannoulias.

**Funding statement:** This study was funded by a competitive grant from ViiV Healthcare. EPPICC receives funding from the Penta Foundation (http://penta-id.org). The MRC Clinical Trials Unit at UCL is supported by the Medical Research Council (programme number: MC_UU_00004/03). The CORISPES study of the clinical-epidemiological analysis of HIV infection in children and adolescents, pregnant women and their children at a national level is supported by the Ministry of Health of Spain (Project 202007PN0002).

### Conflicts of interest

Marisa Luisa Navarro has collaborated in educational activities supported by Gilead, GlaxoSmithKline, MSD, Pfizer, Sanofi Pasteur, Janssen, ViiV Healthcare and as an investigator in clinical trials for GlaxoSmithKline, Janssen, MSD, Pfizer, Sanofi; and as a consultant on GlaxoSmithKline Advisory Boards and research grants of GILEAD, ISCIII and IISGM of Spain. All other authors declare no conflicts of interest.
